# 1-Acetyl-3-[2-(2,3,5,6-tetra­fluoro­phen­yl)hydrazin-1-yl­idene]indolin-2-one

**DOI:** 10.1107/S1600536810022580

**Published:** 2010-06-16

**Authors:** Humayun Pervez, Muhammad Yaqub, Maqbool Ahmad, M. Nawaz Tahir, Robina Akhtar

**Affiliations:** a Department of Chemistry, Bahauddin Zakariya University, Multan 60800, Pakistan; b Department of Physics, University of Sargodha, Sargodha, Pakistan

## Abstract

In the title compound, C_16_H_9_F_4_N_3_O_2_, the dihedral angle between the aromatic ring systems is 4.10 (14)° and a bifurcated intra­molecular N—H⋯(O,F) hydrogen bond generates an *S*(6) ring for the O-atom acceptor and an *S*(5) ring for the F-atom acceptor. A short C—H⋯O conact also occurs. In the crystal, mol­ecules are linked by C—H⋯O inter­actions.

## Related literature

For background on related isatin derivatives, see: Pervez *et al.* (2007 ▶, 2008 ▶, 2010*a* ▶). For related structures, see: Abad *et al.* (2006 ▶); Pervez *et al.* (2010*b* ▶). For graph-set notation, see: Bernstein *et al.* (1995 ▶).
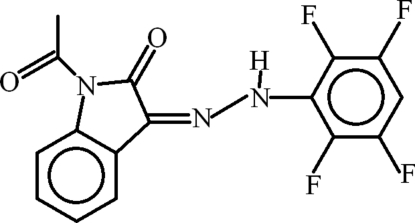


## Experimental

### 

#### Crystal data

C_16_H_9_F_4_N_3_O_2_
*M* *_r_* = 351.26
Monoclinic, 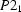

*a* = 9.8993 (19) Å
*b* = 4.7740 (6) Å
*c* = 16.066 (3) Å
β = 104.807 (8)°
*V* = 734.0 (2) Å^3^
*Z* = 2
Mo *K*α radiation
μ = 0.14 mm^−1^
*T* = 296 K
0.32 × 0.24 × 0.22 mm

#### Data collection

Bruker Kappa APEXII CCD diffractometer
Absorption correction: multi-scan (*SADABS*; Bruker, 2005 ▶) *T*_min_ = 0.942, *T*_max_ = 0.952
6095 measured reflections
1462 independent reflections
749 reflections with *I* > 2σ(*I*)
*R*_int_ = 0.087

#### Refinement

*R*[*F* ^2^ > 2σ(*F* ^2^)] = 0.046
*wR*(*F* ^2^) = 0.080
*S* = 0.96
1462 reflections
227 parameters
1 restraint
H-atom parameters constrained
Δρ_max_ = 0.14 e Å^−3^
Δρ_min_ = −0.18 e Å^−3^

### 

Data collection: *APEX2* (Bruker, 2007 ▶); cell refinement: *SAINT* (Bruker, 2007 ▶); data reduction: *SAINT*; program(s) used to solve structure: *SHELXS97* (Sheldrick, 2008 ▶); program(s) used to refine structure: *SHELXL97* (Sheldrick, 2008 ▶); molecular graphics: *ORTEP-3* (Farrugia, 1997 ▶) and *PLATON* (Spek, 2009 ▶); software used to prepare material for publication: *WinGX* (Farrugia, 1999 ▶) and *PLATON*.

## Supplementary Material

Crystal structure: contains datablocks global, I. DOI: 10.1107/S1600536810022580/hb5498sup1.cif

Structure factors: contains datablocks I. DOI: 10.1107/S1600536810022580/hb5498Isup2.hkl

Additional supplementary materials:  crystallographic information; 3D view; checkCIF report

## Figures and Tables

**Table 1:** Hydrogen-bond geometry (Å, °)

*D*—H⋯*A*	*D*—H	H⋯*A*	*D*⋯*A*	*D*—H⋯*A*
N2—H2⋯O2	0.86	1.99	2.694 (5)	139
N2—H2⋯F1	0.86	2.29	2.658 (5)	106
C6—H6⋯O1	0.93	2.33	2.857 (8)	116
C14—H14⋯O1^i^	0.93	2.32	3.217 (7)	163

Symmetry code: (i) 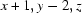.

**Fig. 1.**
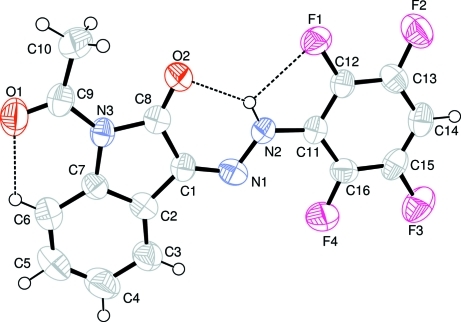
View of (I) with displacement ellipsoids drawn at the 50% probability level. The dotted lines indicate the intra-molecular H-bondings.

**Fig. 2.**
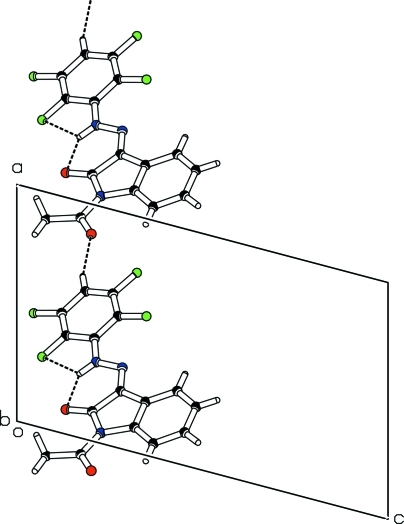
The partial packing of (I), which shows that molecules form one-dimensional polymeric chains extending along the a axis.

## supplementary crystallographic information

### Comment

In continuation of our previous work on the synthesis of isatin derivatives having physiological properties (Pervez *et al.*, 2007, 2008, 2010*a*, 2010*b*), we report herein the synthesis and crystal structure of the title compound (I), (Fig. 1).

The crystal structure of *N*-(2-chloropyrid-4-yl)-*N*^'^-(2,3,5,6-tetrafluorophenyl)urea (Abad *et al.*, 2006) has been published which contains the same flouro substituted phenyl group as in (I).

In (I), the 2-oxoindolin-3-hydrazono group A (N3/C1–C8/O2/N1/N2) and tetrafluorophenyl B (C11—C16/F1–F4) are planar with r. m. s. deviations of 0.0197 and 0.0121 Å, respectively. The dihedral angle between A/B is 4.10 (14)°. The acetyl moiety (O1/C9/C10) is oriented at 6.21 (83)° with its parent group A. One S(5) ring motif (Bernstein *et al.*, 1995) is formed due to intramolecular H-bonding of N—H···F type, two S(6) ring motifs due to N—H···O and C—H···O interactions (Table 1, Fig. 1) are formed. The molecules are stabilized in the form of one dimensional polymeric chains extending along the *a* axis (Fig. 2).

### Experimental

A solution of 1-acetylisatin (0.95 g, 5.0 mmol) in ethanol (50 ml) was added to the solution of 2,3,5,6-tetrafluorophenyl hydrazine (0.90 g, 5.0 mmol) made in concentrated sulfuric acid (8 ml) and diluted with ethanol (50 ml). The reaction mixture was then refluxed for 30 min. The bright yellow crystalline solid formed during refluxing was collected by suction filtration. Thorough washing with hot ethanol furnished the desired compound (I) in pure form (0.40 g, 23%), m.p. 445 K. Bright yellow prisms of (I) were grown in chloroform by slow evaporation method at room temperature.

### Refinement

In the absence of anamolous scattering, the Friedal pairs were merged before refinement. The H-atoms were positioned geometrically (N–H = 0.86 Å, C–H = 0.93–0.96 Å) and refined as riding with *U*_iso_(H) = *xU*_eq_(C, N), where *x* = 1.5 for methyl and *x* = 1.2 for all other H-atoms.

### Crystal data

**Table:**

C_16_H_9_F_4_N_3_O_2_	*F*(000) = 356
*M**_r_* = 351.26	*D*_x_ = 1.589 Mg m^−^^3^
Monoclinic, *P*2_1_	Mo *K*α radiation, λ = 0.71073 Å
Hall symbol: P 2yb	Cell parameters from 749 reflections
*a* = 9.8993 (19) Å	θ = 2.6–25.3°
*b* = 4.7740 (6) Å	µ = 0.14 mm^−^^1^
*c* = 16.066 (3) Å	*T* = 296 K
β = 104.807 (8)°	Prism, yellow
*V* = 734.0 (2) Å^3^	0.32 × 0.24 × 0.22 mm
*Z* = 2	

### Data collection

**Table:**

Bruker Kappa APEXII CCD diffractometer	1462 independent reflections
Radiation source: fine-focus sealed tube	749 reflections with *I* > 2σ(*I*)
graphite	*R*_int_ = 0.087
Detector resolution: 8.10 pixels mm^-1^	θ_max_ = 25.3°, θ_min_ = 2.1°
ω scans	*h* = −11→11
Absorption correction: multi-scan (*SADABS*; Bruker, 2005)	*k* = −5→5
*T*_min_ = 0.942, *T*_max_ = 0.952	*l* = −19→19
6095 measured reflections	

### Refinement

**Table:**

Refinement on *F*^2^	Primary atom site location: structure-invariant direct methods
Least-squares matrix: full	Secondary atom site location: difference Fourier map
*R*[*F*^2^ > 2σ(*F*^2^)] = 0.046	Hydrogen site location: inferred from neighbouring sites
*wR*(*F*^2^) = 0.080	H-atom parameters constrained
*S* = 0.96	*w* = 1/[σ^2^(*F*_o_^2^) + (0.0218*P*)^2^] where *P* = (*F*_o_^2^ + 2*F*_c_^2^)/3
1462 reflections	(Δ/σ)_max_ < 0.001
227 parameters	Δρ_max_ = 0.14 e Å^−^^3^
1 restraint	Δρ_min_ = −0.18 e Å^−^^3^

### Special details

**Table:**

Geometry. Bond distances, angles *etc*. have been calculated using the rounded fractional coordinates. All su's are estimated from the variances of the (full) variance-covariance matrix. The cell e.s.d.'s are taken into account in the estimation of distances, angles and torsion angles
Refinement. Refinement of *F*^2^ against ALL reflections. The weighted *R*-factor *wR* and goodness of fit *S* are based on *F*^2^, conventional *R*-factors *R* are based on *F*, with *F* set to zero for negative *F*^2^. The threshold expression of *F*^2^ > σ(*F*^2^) is used only for calculating *R*-factors(gt) *etc*. and is not relevant to the choice of reflections for refinement. *R*-factors based on *F*^2^ are statistically about twice as large as those based on *F*, and *R*- factors based on ALL data will be even larger.

### Fractional atomic coordinates and isotropic or equivalent isotropic displacement parameters (Å^2^)

**Table:**

	*x*	*y*	*z*	*U*_iso_*/*U*_eq_	
F1	0.3005 (3)	−0.4142 (6)	0.06872 (17)	0.0638 (12)	
F2	0.4748 (3)	−0.8205 (7)	0.0458 (2)	0.0791 (16)	
F3	0.7604 (3)	−0.6503 (7)	0.3236 (2)	0.0843 (14)	
F4	0.5877 (3)	−0.2576 (8)	0.34942 (19)	0.0820 (14)	
O1	−0.1264 (4)	0.8071 (9)	0.2007 (3)	0.0780 (17)	
O2	0.1041 (3)	0.1514 (8)	0.1326 (2)	0.0613 (14)	
N1	0.3432 (4)	0.0297 (9)	0.2843 (3)	0.0520 (17)	
N2	0.3382 (4)	−0.1238 (9)	0.2140 (3)	0.0492 (17)	
N3	0.0446 (4)	0.4800 (9)	0.2270 (3)	0.0472 (17)	
C1	0.2424 (5)	0.2126 (11)	0.2794 (3)	0.045 (2)	
C2	0.2254 (6)	0.3936 (11)	0.3472 (3)	0.048 (2)	
C3	0.3062 (6)	0.4269 (12)	0.4321 (4)	0.067 (3)	
C4	0.2608 (7)	0.6204 (15)	0.4832 (4)	0.085 (3)	
C5	0.1400 (8)	0.7727 (13)	0.4520 (4)	0.082 (3)	
C6	0.0581 (6)	0.7417 (12)	0.3682 (4)	0.064 (3)	
C7	0.1054 (5)	0.5532 (10)	0.3168 (3)	0.046 (2)	
C8	0.1251 (5)	0.2667 (11)	0.2034 (3)	0.047 (2)	
C9	−0.0694 (6)	0.6214 (14)	0.1721 (4)	0.057 (2)	
C10	−0.1145 (5)	0.5317 (13)	0.0791 (3)	0.078 (3)	
C11	0.4358 (5)	−0.3222 (11)	0.2091 (3)	0.0415 (19)	
C12	0.4162 (5)	−0.4726 (11)	0.1331 (3)	0.046 (2)	
C13	0.5063 (6)	−0.6775 (12)	0.1212 (4)	0.055 (2)	
C14	0.6244 (6)	−0.7476 (12)	0.1850 (4)	0.058 (3)	
C15	0.6455 (6)	−0.5977 (13)	0.2589 (4)	0.055 (2)	
C16	0.5565 (6)	−0.3921 (11)	0.2732 (3)	0.052 (2)	
H2	0.26986	−0.09659	0.16945	0.0589*	
H3	0.38710	0.32286	0.45329	0.0808*	
H4	0.31279	0.64831	0.53964	0.1016*	
H5	0.11238	0.90027	0.48823	0.0984*	
H6	−0.02410	0.84251	0.34781	0.0768*	
H10A	−0.19107	0.64643	0.04892	0.1164*	
H10B	−0.14341	0.33917	0.07589	0.1164*	
H10C	−0.03772	0.55230	0.05320	0.1164*	
H14	0.68534	−0.88833	0.17772	0.0692*	

### Atomic displacement parameters (Å^2^)

**Table:**

	*U*^11^	*U*^22^	*U*^33^	*U*^12^	*U*^13^	*U*^23^
F1	0.059 (2)	0.066 (2)	0.058 (2)	0.0084 (17)	−0.0006 (16)	0.0018 (17)
F2	0.096 (3)	0.066 (2)	0.078 (3)	0.010 (2)	0.027 (2)	−0.013 (2)
F3	0.062 (2)	0.101 (3)	0.083 (2)	0.018 (2)	0.006 (2)	0.024 (2)
F4	0.077 (2)	0.098 (3)	0.060 (2)	0.013 (2)	−0.0025 (19)	−0.010 (2)
O1	0.075 (3)	0.076 (3)	0.083 (3)	0.029 (3)	0.020 (2)	0.002 (2)
O2	0.061 (2)	0.070 (3)	0.052 (2)	0.010 (2)	0.013 (2)	−0.010 (2)
N1	0.062 (3)	0.047 (3)	0.048 (3)	−0.006 (3)	0.016 (2)	−0.002 (3)
N2	0.042 (3)	0.055 (3)	0.048 (3)	0.008 (2)	0.007 (2)	−0.001 (3)
N3	0.048 (3)	0.039 (3)	0.055 (3)	0.008 (2)	0.014 (3)	0.006 (2)
C1	0.048 (4)	0.038 (4)	0.051 (4)	0.003 (3)	0.015 (3)	0.008 (3)
C2	0.058 (4)	0.043 (4)	0.045 (4)	−0.004 (3)	0.018 (3)	0.002 (3)
C3	0.075 (4)	0.069 (5)	0.055 (4)	0.010 (4)	0.011 (4)	−0.004 (4)
C4	0.105 (6)	0.083 (6)	0.062 (5)	0.003 (5)	0.014 (4)	−0.015 (4)
C5	0.112 (6)	0.068 (5)	0.073 (5)	0.001 (5)	0.035 (4)	−0.020 (4)
C6	0.067 (4)	0.056 (4)	0.074 (5)	0.007 (3)	0.027 (4)	−0.003 (4)
C7	0.053 (4)	0.037 (4)	0.050 (4)	−0.004 (3)	0.016 (3)	−0.001 (3)
C8	0.049 (4)	0.042 (4)	0.051 (4)	−0.003 (3)	0.017 (3)	0.006 (3)
C9	0.051 (4)	0.057 (4)	0.066 (4)	0.005 (3)	0.022 (3)	0.008 (3)
C10	0.072 (4)	0.095 (5)	0.057 (4)	0.019 (4)	0.000 (3)	0.000 (4)
C11	0.040 (3)	0.037 (3)	0.051 (4)	−0.001 (3)	0.018 (3)	0.010 (3)
C12	0.046 (4)	0.040 (4)	0.052 (4)	0.006 (3)	0.013 (3)	0.006 (3)
C13	0.068 (4)	0.045 (4)	0.055 (4)	0.001 (3)	0.020 (4)	−0.002 (3)
C14	0.057 (4)	0.044 (4)	0.078 (5)	0.008 (3)	0.028 (4)	0.012 (4)
C15	0.044 (4)	0.057 (4)	0.064 (4)	0.009 (3)	0.012 (3)	0.021 (4)
C16	0.051 (4)	0.056 (4)	0.048 (4)	−0.009 (3)	0.013 (3)	−0.006 (3)

### Geometric parameters (Å, °)

**Table:**

F1—C12	1.362 (6)	C4—C5	1.380 (10)
F2—C13	1.356 (7)	C5—C6	1.391 (9)
F3—C15	1.354 (7)	C6—C7	1.382 (8)
F4—C16	1.347 (6)	C9—C10	1.508 (8)
O1—C9	1.203 (8)	C11—C16	1.404 (7)
O2—C8	1.233 (6)	C11—C12	1.387 (7)
N1—N2	1.337 (6)	C12—C13	1.370 (8)
N1—C1	1.313 (7)	C13—C14	1.385 (9)
N2—C11	1.370 (7)	C14—C15	1.356 (9)
N3—C7	1.457 (7)	C15—C16	1.377 (8)
N3—C8	1.404 (7)	C3—H3	0.9300
N3—C9	1.414 (8)	C4—H4	0.9300
N2—H2	0.8600	C5—H5	0.9300
C1—C8	1.477 (7)	C6—H6	0.9300
C1—C2	1.434 (7)	C10—H10A	0.9600
C2—C7	1.391 (8)	C10—H10B	0.9600
C2—C3	1.403 (8)	C10—H10C	0.9600
C3—C4	1.385 (9)	C14—H14	0.9300
			
N2—N1—C1	116.8 (4)	F1—C12—C13	119.4 (5)
N1—N2—C11	123.6 (4)	C11—C12—C13	122.9 (5)
C7—N3—C8	108.8 (4)	F1—C12—C11	117.7 (4)
C7—N3—C9	124.4 (4)	C12—C13—C14	121.7 (5)
C8—N3—C9	126.6 (5)	F2—C13—C12	118.4 (5)
N1—N2—H2	118.00	F2—C13—C14	119.9 (5)
C11—N2—H2	118.00	C13—C14—C15	115.6 (6)
N1—C1—C2	126.2 (5)	F3—C15—C16	116.9 (5)
N1—C1—C8	126.2 (5)	C14—C15—C16	124.1 (6)
C2—C1—C8	107.6 (4)	F3—C15—C14	119.1 (5)
C1—C2—C3	131.2 (5)	C11—C16—C15	120.5 (5)
C3—C2—C7	120.2 (5)	F4—C16—C11	120.5 (5)
C1—C2—C7	108.6 (4)	F4—C16—C15	118.9 (5)
C2—C3—C4	117.5 (6)	C2—C3—H3	121.00
C3—C4—C5	121.2 (6)	C4—C3—H3	121.00
C4—C5—C6	122.2 (6)	C3—C4—H4	119.00
C5—C6—C7	116.4 (6)	C5—C4—H4	119.00
C2—C7—C6	122.5 (5)	C4—C5—H5	119.00
N3—C7—C2	108.4 (4)	C6—C5—H5	119.00
N3—C7—C6	129.1 (5)	C5—C6—H6	122.00
N3—C8—C1	106.5 (4)	C7—C6—H6	122.00
O2—C8—N3	126.9 (5)	C9—C10—H10A	110.00
O2—C8—C1	126.6 (5)	C9—C10—H10B	109.00
O1—C9—N3	119.4 (6)	C9—C10—H10C	109.00
O1—C9—C10	122.6 (6)	H10A—C10—H10B	109.00
N3—C9—C10	118.0 (5)	H10A—C10—H10C	109.00
N2—C11—C12	117.8 (4)	H10B—C10—H10C	109.00
C12—C11—C16	115.1 (5)	C13—C14—H14	122.00
N2—C11—C16	127.1 (5)	C15—C14—H14	122.00
			
C1—N1—N2—C11	179.8 (5)	C1—C2—C7—C6	177.8 (5)
N2—N1—C1—C2	178.5 (5)	C3—C2—C7—N3	178.8 (5)
N2—N1—C1—C8	−0.7 (8)	C3—C2—C7—C6	−2.1 (8)
N1—N2—C11—C12	178.2 (5)	C2—C3—C4—C5	0.6 (10)
N1—N2—C11—C16	−2.2 (8)	C3—C4—C5—C6	−0.3 (11)
C8—N3—C7—C2	2.2 (6)	C4—C5—C6—C7	−1.2 (10)
C8—N3—C7—C6	−176.7 (5)	C5—C6—C7—N3	−178.8 (5)
C9—N3—C7—C2	−172.7 (5)	C5—C6—C7—C2	2.4 (8)
C9—N3—C7—C6	8.4 (8)	N2—C11—C12—F1	−0.5 (7)
C7—N3—C8—O2	179.4 (5)	N2—C11—C12—C13	−179.4 (5)
C7—N3—C8—C1	−2.2 (5)	C16—C11—C12—F1	179.8 (4)
C9—N3—C8—O2	−5.9 (9)	C16—C11—C12—C13	0.9 (8)
C9—N3—C8—C1	172.6 (5)	N2—C11—C16—F4	−0.6 (8)
C7—N3—C9—O1	−3.7 (9)	N2—C11—C16—C15	180.0 (5)
C7—N3—C9—C10	176.2 (5)	C12—C11—C16—F4	179.1 (5)
C8—N3—C9—O1	−177.7 (5)	C12—C11—C16—C15	−0.4 (8)
C8—N3—C9—C10	2.3 (8)	F1—C12—C13—F2	−1.6 (8)
N1—C1—C2—C3	0.5 (10)	F1—C12—C13—C14	−179.1 (5)
N1—C1—C2—C7	−179.4 (5)	C11—C12—C13—F2	177.3 (5)
C8—C1—C2—C3	179.8 (6)	C11—C12—C13—C14	−0.2 (9)
C8—C1—C2—C7	0.0 (6)	F2—C13—C14—C15	−178.4 (5)
N1—C1—C8—O2	−0.8 (9)	C12—C13—C14—C15	−0.9 (9)
N1—C1—C8—N3	−179.3 (5)	C13—C14—C15—F3	−178.6 (5)
C2—C1—C8—O2	179.9 (5)	C13—C14—C15—C16	1.5 (9)
C2—C1—C8—N3	1.4 (6)	F3—C15—C16—F4	−0.2 (8)
C1—C2—C3—C4	−179.3 (6)	F3—C15—C16—C11	179.3 (5)
C7—C2—C3—C4	0.6 (9)	C14—C15—C16—F4	179.7 (5)
C1—C2—C7—N3	−1.3 (6)	C14—C15—C16—C11	−0.9 (9)

### Hydrogen-bond geometry (Å, °)

**Table:**

*D*—H···*A*	*D*—H	H···*A*	*D*···*A*	*D*—H···*A*
N2—H2···O2	0.86	1.99	2.694 (5)	139
N2—H2···F1	0.86	2.29	2.658 (5)	106
C6—H6···O1	0.93	2.33	2.857 (8)	116
C14—H14···O1^i^	0.93	2.32	3.217 (7)	163

Symmetry codes: (i) *x*+1, *y*−2, *z*.
